# What, Me Worry? Research Policy and the Open Embrace of Industry-Academic Relations

**DOI:** 10.3389/frma.2021.600706

**Published:** 2021-05-28

**Authors:** Bennett Holman

**Affiliations:** ^1^ Yonsei University, Seoul, South Korea; ^2^ University of Johannesburg, Johannesburg, South Africa

**Keywords:** industry-funded science, academic engagement, philosophy of science, policy research, commodification of knowledge, feminist epistemology, regulatory science

## Abstract

The field of research policy has conducted extensive research on partnerships between industry and academics and concluded that such collaborations are generally beneficial. Such a view stands in stark contrast to the literature in the philosophy of science which almost wholly finds such collaborations corrosive to scientific inquiry. After reviewing the respective literatures, I propose explanations for these polarized views which support the claim that both disciplines have only a partial vantage point on the effects of industry-funded science. In closing, I outline how the research agendas of each discipline might remediate their respective shortcomings.

## Introduction

It should not be surprising to find that when different academic disciplines study the same topic matter that different aspects of a phenomenon come to the fore, especially when that phenomenon is a complex human institution. Nevertheless, for the scholar immersed in her own way of conceptualizing a phenomenon, it is disorienting to encounter another framework. It is like stepping into a similar but parallel universe in which familiar objects are cast in a different light and aspects of reality which had faded into the background and which had been taken for granted, now come into sharp relief against unexpected absences.

Such is the case in the study of industry-funded science as seen from the vantage points of philosophy of science and from science policy studies. While both disciplines have an extensive literature on the influence of industry-funding on science, they have remained, so far as I can discern, almost completely distinct. To wit, review articles of academic-industry relations summarizing research in science policy (Perkmann et al., 2021) and philosophy of science (Holman and Elliott, 2018) do not share a single common source despite both including over 100 citations. Of course, some of the sources in the former were published after 2018 and could not have been cited in the latter, but this does not explain the absence of the research cited in the philosophy of science review from informing the science policy literature. In short, there really are two largely independent bodies of research.

Accordingly, the primary function of this paper is to begin put these two literatures into contact with one another, to identify areas of overlap, and to suggest how each could draw most fruitfully from the other. I first review the literatures in philosophy of science (*The Perils of Industry Funding in Science*) and research policy (*Moderating Industry Collaborations and Maximizing Scientific Output*). In *Two Worlds*, I confront the drastically different attitudes that each discipline takes towards the influence of industry. I argue that notwithstanding the wealth of scholarship which philosophers of science could profitably draw from, that the science policy literature lacks the fundamental conceptual resources to gauge the epistemic impact of industry on the scientific process and thus their rosy view of industry-funded science stems from a blind spot rather than a superior vantage point. I conclude by identifying areas of overlap and ways in which research in each discipline may benefit from incorporating the research done in the other.

## The Perils of Industry Funding in Science

Philosophers of science are standardly interested in the fundamental questions that underpin scientific inquiry. This includes both the central concepts deployed within science (e.g., causation, explanation, etc., ) as well as a concern for the method(s) and overall reliability of a science generally. Though it became better integrated with the history of science over the course of the 20th century, work in philosophy of science generally remained removed from science in practice. In the early 20th century the social epistemology of science has emerged within philosophy of science as an attempt to situate traditional philosophical concerns within a contextualized and grounded account of inquiry (Goldman, 1999; Solomon, 2001; Longino, 2002). Attention to industry funding and science has only begun to attract sustained attention within the past decade.^1^

With some exceptions (e.g., Adam et al., 2006), the primary focus has been on how industry-funded science distorts or corrupts the scientific endeavor. In stark contrast, the science policy literature generally regards the influence of the private sector with something between neutrality and unabashed enthusiasm. In this section and the next, I briefly survey the respective literatures as means of illustrating the difference in foci and to substantiate the claim that there is a stark difference between the way that industry is regarded.

Holman and Elliott (2018) organize the philosophy of science literature schematically according to the ways in which industry can distort various stages of inquiry.^2^ At the most fundamental level, industry can shape the concepts scientists work with in ways that predispose inquiry to reach commercially favored outcomes. One prominent manifestation of this in the medical field is disease mongering—or the pathologization of normal human suffering in order to increase the potential commercial applications of drugs (Brown, 2002; Moynihan and Cassels 2005; González-Moreno,et al., 2015). Another means of shaping the communal body of knowledge is to channel research by selectively funding projects with commercially advantageous outcomes and away from establishing facts that would be economically damaging. In so doing, industry is not simply funding one line of research over another, they are actively preventing the scientific community—and thus the general public—from coming to know something which would be in their objective interest to learn. Such active maintenance of ignorance has now formed its own research domain under the label of agnotology (Proctor, 2011; Fernandez Pinto, 2015; 2017).

Even when threatening questions must be asked (for example when they are required to satisfy regulatory approval), industry often works assiduously to make sure that the methods, experimental design, and statistical analysis used to answer those questions yield commercially favorable outcomes (Steele, 2018; Stegenga, 2018). Similarly, a wide latitude exists on how results are discussed, which opens up the door for a considerable degree of rhetorical spin (Biddle, 2007; Matheson, 2008). If all else fails, undesirable results can simply be withheld from publication (McGarity and Wagner, 2008; Jukola, 2015a).

Finally, philosophers of science have contended that to understand the influence of industry on science, that the focus must ultimately move beyond the individual to include the larger social structure within which science operates (Biddle, 2007; Wilholt, 2009; Intemann and de Melo-Martín, 2014; Holman, 2015, 2019). Following the lead of the tobacco Industry, numerous sectors (e.g., petroleum, pharmaceuticals, lead, etc.) have used high level strategies to manipulate scientific knowledge (McGarity and Wagner, 2008; Michaels 2008; Oreskes and Conway, 2010; White and Bero, 2010). Understanding the larger social context is necessary both because some effects only occur at the social level (Holman and Bruner, 2017) and because solutions to active manipulation must consider a full range of how industry would attempt to circumvent reform in order to increase the likelihood that it will be effective (Holman and Geislar, 2018).

## Moderating Industry Collaborations and Maximizing Scientific Output

The discipline of policy research is populated primarily by scholars in either economics departments or business schools.^3^ They take their audience to be two-fold. First, it aims to be of practical importance for university managers and government policy makers. Indeed, the size of this audience is growing because of increased pressure from national governments to demonstrate that recipients of research funding make a demonstrable public impact (Sá et al., 2013). In addition, numerous nonprofit and government agencies (e.g., the Gates Foundation, The Wellcome Trust, the President’s Council of Advisors on Science and Technology, the Food and Drug Administration) are encouraging collaborations between industry and academia (Drazen, 2015). Indeed, over the past decade the entire pharmaceutical industry has restructured a significant portion of their research and development into university-industry partnerships (Robinson, 2019; 2020). On this front research policy is supposed to inform and facilitate successful engagement.

Beyond this, the university is a readily available social system which has a long history of scholarly study (e.g., Merton, 1973). As Perkmann et al. (2021) note, exploring the interface of industry and university research has provided an opportunity for scholars to study norms around information sharing and the violation thereof (Haas and Park, 2010): how do hybrid organizations manage the demands? Are there disparate practices and self-identities of two conflicting social institutions (Sauerman and Stephan, 2013; Perkmann, et al., 2019)? And which factors affect researchers’ uptake of new practices involving technology transfers (Bercovitz and Feldman, 2007). On this front, the interface of science and industry is of interest to, and can potentially draw from, a wide range of social scientific frameworks.

Collectively, the reviews by Perkmann et al. (2013, 2021) cover thirty years of research on what they call “academic engagement.” Strictly speaking, academic engagement is narrower than industry-funded science. It is meant to encapsulate instances of university researchers interacting with industry (e.g., collaborative research, contract research, consulting). For the moment, it is important to note that academic engagement does not include science conducted exclusively in-house in private corporations, the work of industry-funded think tanks, nor does it include “commercialization” which is designated as the creation of intellectual property or founding a for-profit business from one’s academic work. I will return to these distinctions in *Two Worlds* and discuss the extent to which this shapes the respective literatures.

While it is important to foreground that Perkmann et al. (2013, 2021) are considering a narrower range of phenomena, there is a considerable degree of conceptual overlap in the areas of study. Both reviews are primarily focused on what effects industry involvement has on scientific inquiry. Perkmann et al.‘s review is organized by describing what factors make a researcher more likely to participate in academic engagement and then shifts to outlining the consequences for academic research and the commercial consequences of academic engagement. Those uninterested in the determinants of engagement may wish to skip to *The Consequences of Engaging with Industry* where I discuss the research on its consequences.

### The Determinants of Engaging With Industry

At least in the United Kingdom, men are more likely to participate in academic engagement than women (Abreu and Grinevich, 2013; 2017); however, there were some specific activities (public engagement and informal advice) in which women were more likely than men to engage in (Lawson, et al., 2016). Moreover, when universities had systematic policies to promote women’s careers, these differences were significantly reduced (Tartari and Salter, 2015). There are mixed effects on whether older academics are more likely engaged with industry irrespective of whether it is measured by biological age (Tartari and Breschi, 2012; Abreu and Grinevich, 2013; Lawson, et al., 2019; Iorio, et al., 2017) or in years since PhD (Schuelke-Leech, 2013; Acshhoff and Grimpe, 2014; Huyghe and Knockaert, 2015). However, there is a clear positive relation with professional rank obtained (i.e., from research assistant to full professor (Tartari and Breschi, 2012; Abreu and Grinevich, 2013; Lawson, et al., 2019)).

Prior experiences also have an impact on likelihood of engaging with industry. Tartai et al. (2012) find academics that have previously worked outside of academia perceive fewer barriers to academic engagement. Indeed, such researchers are more likely to engage with industry (Abreu and Grinevich, 2013) even if that experience is in the non-profit sector (Gulbrandsen and Thune, 2017). Once an academic has participated in some form of academic engagement, most will do so again (Lawson, et al., 2016).

Other research has focused on the academic profile of those inclined towards collaborating with industry. Such researchers tend to be more prolific publishers (Aschhoff and Grimpe, 2014; D’Este et al., 2019; Ding and Choi, 2011), but are not more likely to publish work of superior quality (Ding and Choi, 2011; Tartari et al., 2014; Zi and Blind 2015). Unsurprisingly, researchers who engage with industry are more likely to publish in applied scientific journals (Tartari and Breschi, 2012; Zi and Blind, 2015).

A researcher’s context also had a significant effect. Academics in departments where their colleagues were engaged with industry were more likely to do so themselves (Aschhoff and Grimpe 2014; Tartari et al., 2014). University policies also have an effect. When universities have stricter policies about disclosure of conflicts of interest, researchers are less likely to engage with industry (Halilem et al., 2017). If a university takes a higher percentage for royalties for work done at the institution, researchers tend to shift towards engagement (e.g., consulting) and away from developing their own intellectual property (Halilem et al., 2017).

Finally, in terms of consciously held, individual motivations, policy research breaks up the conceptual terrain into intellectual challenge (“puzzles”), professional recognition (“ribbons”), and personal financial gain (“gold” (Stephan and Levin, 1992; Lam, 2011)). When asked why they engaged with industry, ribbons and puzzles emerged as the primary motivations (Lam, 2011). This finding was refined in German academics, standardization efforts were primarily motivated by a desire to solve puzzles, while patenting was driven by gold (Blind et al., 2018). Yet this framework does not capture the full range of motivations to engage with industry. Italian and Spanish researchers cite obtaining research funding—rather than personal gain—as their primary motivation, though they express concern that such interactions may limit their academic freedom and tarnish their reputation (Tartari and Breschi, 2012; Ramos-Vielba et al., 2016). Iorio et al. (2017) unpack the desire to obtain research funding, finding that it is driven by a desire to benefit society rather than generating new knowledge. This finding departs from research from the United Kingdom and Denmark which finds that increasing knowledge is a major driver of engagement (Hughes et al., 2016; Kongsted et al., 2017).

### The Consequences of Engaging With Industry

In policy research, the consequences of engagement are primarily framed in terms of quality and quantity of subsequent research as measured by the number of publications the author appears on and the ranking of the journal where the articles are published (e.g., as measured by impact factor). By these measures, academic engagement leads researchers to produce both a higher quantity of research (Hottenrott and Lawson, 2017; Garcia et al., 2020) and a higher caliber of research (Hottenrott and Lawson, 2017). Especially large productivity gains are observed when researchers are selective about who they partner with (Callaert et al., 2015). However, other research has suggested factors that modify the effect of engagement on productivity. In particular, Banal-Estañol et al. (2015) found that engagement tended to increase productivity, however, once academics began engaging with industry in more than 30–40% of their projects, productivity decreased because “research ideas may then be of lower value, industry may impose non-disclosure clauses or because extensive collaboration could reduce the time to do research and cause attention problems” (p. 1173). Moreover, it appears that not all forms of engagement (e.g., consulting) increase productivity (Rentocchini et al., 2014).

Beyond publication, receiving funding from industry has been shown to increase secrecy. Researchers who received industry funding were twice as likely to deny requests to share data or other research methods and materials, as well as to delay publication of their findings (Czarnitzki et al., 2015a; Czarnitzki et al., 2015b). Nevertheless, academic engagement has been found to increase researchers’ reputation amongst their peers (Hughes, et al., 2016). Perhaps because industry engagement serves as a ribbon, Fini, et al. (2018) find that moderate engagement with industry increases both a researcher’s reputation and ability to obtain public grant funding. However, at high levels of engagement they find that a researcher’s reputation amongst their peers decreases as they begin to suffer an identity penalty (viz. they start to be viewed as an industry researcher as opposed to an academic researcher who occasionally partners with industry).

In terms of their commercial output, engagement with industry increases a researcher’s patent output (Beaudry and Kananian, 2013; Lawson, 2013; Libaers, 2017; cf.; Bikard et al., 2019), though such researchers are also more likely to circumvent the universities technology transfer office (Perkmann et al., 2015; Goel and Göktepe-Hultén, 2018). As with previous findings, the result is curvelineal (e.g., at very high levels engagement decreases patent output). Finally, serving as a company’s scientific adviser has been shown to decrease the likelihood of starting one’s own company (Fritsch and Krabel, 2012). On these grounds, Perkmann et al. (2021) assure policy makers that “academic engagement is complementary with research, practiced by scientifically productive individuals … and likely to have positive effects on research productivity and other research related performance measures” (p. 4).

## Two Worlds

I find it difficult to keep in mind that these two literatures are about the same substantive topic (I hope readers now feel this way too). Having summarized both bodies of research, I wish to: (1) suggest a reason why the research policy literature is predominantly positive on academic-industry partnerships (*Upon the Altar of Productivity*); (2) explore why philosophy of science is predominately negative (*The View From Somewhere*); and (3) identify some areas where these literatures might begin to inform one another (Discussion: Synergy or Schism). I wish to be clear that I do not pretend that the explanations I offer are “the” explanations, indeed, I won’t even offer the same type of explanation in both cases. Rather, my only contention is that the explanations shed some light on the phenomenon, why each discipline generates a one-sided account of industry academic partnerships, and how their respective research programs might move forward.

### Upon the Altar of Productivity

Perhaps the most significant difference between the two accounts is that policy research is a social science. To be clear, both literatures are clearly empirical in some broad sense as the philosophical literature is heavily based on particular episodes of scientific inquiry. Nevertheless, policy research, at least insofar as it is captured by Perkmann et al. (2013, 2021), is fundamentally rooted in quantitative research methods in ways that predispose it to take the functioning of science at face value. While it is clear that there are also likely to be structural reasons why economists and business professors are less critical of academics collaborating with profit-seeking entities than philosophers, I want to focus on the difficulty of detecting the deleterious effect of industry, given the outcome variables policy researchers are inclined to collect.

Consider, for example, a recent high-profile case of academic engagement in the study of remdesivir for the treatment of COVID-19 (Beigel et al., 2020). The trial was primarily funded by the American government, but employees of Gilead Sciences (the manufacturers remdesivir) “participated in discussions about protocol development and in weekly protocol team calls” (p. 11). Moreover, numerous authors on the publication had some form of previous engagement with Gilead (e.g., consulting). The article was published in the *New England Journal of Medicine* one the most elite medical journals in the world and in the first three months since its publication it has garnered over 500 citations. Practically speaking, it instantly changed medical practice worldwide. From a research policy perspective this appears to be a clear triumph.

Yet surely, in some very important sense, this research can be considered a success only if remdesivir is in fact an effective treatment for COVID-19 (or at least if the trial was “fair test” (Evans, et al., 2011)). But this depends on a number of substantive and methodological questions. For example, the trial was stopped early because the results were significant on the primary end point (time to recovery), was it appropriate to stop the trial at the point when significance had been reached? Did the statistical analyses used properly take into account the fact that interim data was being analyzed with the possibility of terminating the trial? Was the published primary outcome measure appropriate? Was the decision to change the primary outcome measure during the trial appropriate? And so on.

In short, answering questions about the integrity of any particular piece of research is going to require a considerable amount of time. Current studies on research output take as data hundreds or thousands of researchers each with tens or hundreds of articles. There is no clear practicable way to exercise anything close to the level of scrutiny that would seem to be required to independently assess each article of each author. Even if there were, doing so would require a considerable amount of expert knowledge, which even if one had in some particular domain, would be required in every academic domain and sub-domain under study.

Using quantified outcome variables makes it possible for a reasonably small group of researchers to assess wide swathes of academia without needing to understand the content of the subject field that they are studying. Moreover, their approach to doing so mirrors a logic that is seemingly practical and familiar. For example, when a department seeks to hire a new position, it is often the case that they are hiring someone to fill a gap in the intellectual breadth of the department. That is, the very reason that they are hiring is because the current faculty lack someone with the very expertise that would be needed to independently assess the academic qualifications of the candidates under consideration. In such circumstances, a natural shorthand for assessing candidates is to assume that a publication is a genuine indication that the candidate has contributed to that area of knowledge. Similarly, since most fields have journals with a hierarchical system of prestige, one assumes that an article published in an elite journal is of higher quality than one published in a smaller specialty journals. In short, if you are willing to assume that a discipline is well-functioning, publication record is an accurate, though impoverished, proxy for merit and it is difficult to see how one could reasonably discard this as a simplifying assumption and continue to carry out traditional research policy projects.

Such problems are amplified when it comes to assessing if industry engagement biases the direction of the research agenda. As Inmaculada de Melo-Martin (2019) has pointed out, “it is not clear that there is any such thing as the *epistemically correct* research agenda or the *epistemically appropriate* direction for a research agenda to take” (p. 8, emphasis in original). Indeed, it has long been argued that choices about what research to undertake are underdetermined (e.g., Kuhn, 1962; Lakatos, 1970). Nevertheless, even if there is no uniquely correct course of research, this does not necessarily imply that anything goes. There may well be some research agendas which are objectively deleterious (e.g., the tobacco industry’s funding of research that questioned the link between smoking and cancer). Yet the ability to assess each individual’s choice in research agenda, because there are even more degrees of freedom than in research design, would be correspondingly more knowledge intensive than scrutinizing the quality of research.

In sum, given the goal to assess a large heterogeneous collection of academic disciplines by researchers who will generally lack the subject area expertise necessary to make independent judgments, the discipline of policy research has coalesced to using easily accessible, quantitatively tractable outcome measures. While these metrics may be crude, in a well-functioning discipline, such metrics might be a reasonable proxy for scientific contribution. Moreover, given the aims of the discipline to serve university managers, these measures of productivity might be the relevant variable to study irrespective of their validity. To the extent that managers aim to burnish the image of their institution and external bodies (e.g., QS world rankings) use these measures to evaluate universities, tracking these metrics may well be instrumentally rational. Nevertheless, the principle of charity would dictate that managers use these metrics not just to manage the university’s reputation, but because they trust that the measures accurately reflect genuine scientific contribution. Similarly, while it may be the case that given their disciplinary aims, research policy scholars are simply not interested in detecting distortions in scientific research caused by industry-funding, a more charitable explanation is that their standard outcome variables preclude such questions from being meaningfully raised.

### The View From Somewhere

Among academic disciplines that make the scientific process a focus of study, philosophy of science has been a relative late comer in its attention towards industry involvement. I hope to show that a brief genealogy of this development fruitfully contextualizes the philosophical literature. I propose that three intellectual antecedents of this emerging body of work can be found, which account for why philosophy of science has largely focused on the perils of industry funding. The first is through feminist epistemology, the second is a focus on areas of science that intersect with regulatory issues, and the third is through concern with the changing nature of the university as an institution.^4^

At the outset it is worth noting that there is nothing inherent in the philosophical approach that should restrict it to abstracting away from the context in which science is conducted. As Heather Douglas (2014) has argued, philosophy’s focus on “the logic of science” is the outcome of a struggle between John Dewey and Bertrand Russell. Russell worried that a focus on utility and application would lead science away from the pure pursuit of knowledge and towards a complicity in the type of destruction that scientists had facilitated over the course of WWI (e.g., gas warfare). Faced with a similar concern, Dewey attributed such evils to a lack of knowledge of what was needed to serve the public good. Indeed, he viewed the very idea of pure science as part of a mythology that facilitated scientists’ ignorance of their responsibility to consider the social consequences of their work.

In part because drawing a sharp distinction between science and values forestalled Marxist critiques of scientific inquiry, Russell’s focus on a pure logic of science dominated American and British philosophy of science and ultimately set the agenda for the next 50 years. As a result, mid-century philosophers of science focused on disembodied questions such as the logic of causation, what constitutes an explanation, and the nature of scientific mechanisms (for example consider anthologies (Curd and Cover, 1998; Boyd et al., 1991, etc.. ). The philosophical debates on these topics grew removed from actual practice.

For example, consider Bas Van Fraassen (1980) fable of the “Tower and the Shadow” in the context of debates surrounding Hempel’s DN model of explanation (Hempel and Oppenheim, 1948). In essence, the DN model considers a scientific explanation as a derivation of an observation from a set of initial conditions and the laws of the relevant science. A common type of objection is that it seems that while the height of a tower could be predicted from the laws of optics and the length of a shadow, the length of a shadow does not explain the height of the tower (Bromberger, 1960). Van Fraassen responds to this objection in his pragmatic account of explanation, arguing that whether it is an explanation depends on contextual factors. In the Tower and the Shadow parable, Van Frassen considers a case where a tower was built to cast a shadow in a particular place at a particular time. Van Frassen (1980) claims that the length of the shadow plus the laws of optics would be satisfactory explanation of the height of the tower in this case. Clearly, such an argument is not grounded on an in-depth study of explanation in the scientific literature.

Even amongst philosophers most immersed in the practice of science, the economics of its practice was nowhere to be found. For example, Karl Popper advocated for an understanding of the historical canon that situated philosophers in their larger societal and historical contexts. Specifically, he argued that it is necessary to study the history of science and mathematics because traditional philosophical problems arise out of “urgent and concrete problems, problems which they found could not be dismissed” (Popper, 1963, p. 73). According to Popper’s account, Plato’s philosophy stems from wrestling with the irrationality of the square root of two from within a Pythagorean framework (that asserted essence of reality is numerical) and Kant’s *Critique of Pure Reason* is an attempt to understand how it was possible for Newton to have discovered the truths of physics. What they show, Popper argues, is that “*genuine philosophical problems are always rooted in urgent problems outside of philosophy* … What matters is not methods or techniques, but a sensitivity to problems and a consuming passion for them; or as the Greeks said, the gift of wonder” (Popper, 1963, p. 72, italics in original). Yet when it came to his conceptualization of science, Popper (1970) regarded it as “subjectless” and nothing but a “system of theories.” Even Kuhn (1962, p. x), whose philosophy of science was richly informed by the history of science, noted the partiality of the view he offered in *The Structure of Scientific Revolution*: “More important, except in occasional brief asides, I have said nothing about the role of technological advance or of external social, economic, and intellectual conditions in the development of the sciences.” It is only recently that the influence of industry on science has found a comfortable home within philosophical discourse.

#### The Feminist Critique

Feminist epistemology of science is the first stream which informs the philosophical discussion. While there are numerous aspects to this body of work, one central theme is that sexist values are subtly—or not so subtly—influencing scientific research. For example, consider the study of primate sexual behavior in langurs by Sarah Blaffer Hrdy. In langur troops, there are periodic bouts of infanticide and Hrdy’s earlier work established that they were evolutionarily rational. Such work extended the Bateman-Trivers paradigm which argued that the sex which physically cared for the offspring most would be a site of resource competition for the other sex. Accordingly, Hrdy (1974) showed that when a new male arrives from outside the troop, killing the troop’s infants is evolutionarily rational because it brings their mothers into estrus sooner and increases the male’s reproductive fitness.

However, inspired by the contemporary feminist movement, Hrdy began to see this account as only half the story. The behavior is evolutionarily rational for the new invading male, but it is clearly not rational for the troop’s females to have their infants killed every 2–3 years, so why did female langurs seem to put up with such behavior? This question led to many others and forced her to reconceptualize old observations. For example, it suggested an explanation for why pregnant langurs would solicit sexual pairings with males outside their troop, an otherwise costly behavior with no obvious reproductive benefit (Hrdy, 1977). It also suggested other meta-scientific questions, such as why females were seen as coy (sexually discriminating) despite the fact that they were actually engaging in a significant amount of sexually promiscuous non-monogamous behavior (Hrdy, 1986/2006).

The answer to the former question is of primary interest to evolutionary biology, the latter question is of primary interest to feminist epistemology. Hrdy’s work was seen by feminists (and by evolutionary biologists as well) as epistemically superior to the work that preceded it. Yet divisions emerge among feminist epistemologists as to what accounts for the superiority.

Feminist empiricists (e.g., Longino, 1990, 2002; Solomon, 2001) have argued that for an account for the superiority of such knowledge one has to analyze the social structure of science. For example, on Longino’s account objective knowledge arises from a properly structured society of diverse inquirers. When a scientific group is homogenous, their values—and the way that those values influence inquiry—go unexamined. In the case of Hrdy, we can see how the Victorian ideal of a sexually chaste female choosing amongst her suitors is replicated in the Bateman-Trivers paradigm. When considering her own early work Hrdy describes how the existing biases within the field shaped her understanding of mating behaviors and produced a dearth of scholarship on female mating strategies: “because theoretically the phenomenon [female promiscuity] should not have existed and therefore there was little theoretical infrastructure for studying it, certainly not the sort of study that could lead to a PhD (or a job)” (p. 135). On Longino’s account, what occurs with the introduction of Hrdy (and other feminists) into the field is that Victorian values are questioned and the ways in which they bias research are exposed and corrected. Objectivity emerges socially out of a clash of subjectivities.

An alternative account of the superiority of Hrdy’s work arises from the work of standpoint theorists (e.g., Collins, 1990; Harding, 1991). On such accounts it is crucially important that Hrdy is a feminist in a patriarchal society. According to standpoint theories, social location systematically influences knowledge production and knowledge systems tend to embed the interests of dominant groups. Because their interests diverge, subjugated groups often have their own standpoint from which to understand the relevant phenomenon. On some accounts (e.g., Hartsock, 1983), power differentials produce subjugated groups with a privileged epistemic position. This occurs because subjugated groups must often be conversant in both their understanding of a phenomenon and the understanding of the dominate groups. Conversely, the dominant group can safely remain ignorant of the subjugated group’s understanding. On other accounts (e.g., Wylie, 2003; Harding, 2004), an epistemically superior standpoint is differentiated from a person’s individual experiences. A standpoint is not simply the experiences of someone from a marginalized group, but is rather a group achievement that arises from the attainment of a “critical consciousness”—an awareness of how power structures have influenced the dominant ideology.

If we return to Hrdy, we might note that the Bateman-Trivers paradigm assumes that nearly all females mate and so do not have a significant fitness differential for evolution to act upon. In adopting the perspective of the female as a site of evolutionary action, Hrdy was breaking significant ground and the fact that she did so was not incidental to her social location and interaction with feminist thought:

In my own case, changes in the way I looked at female langurs were linked to a dawning awareness of male–female power relationships in my own life, though ‘‘dawning’’ perhaps overstates the case … Each step in understanding what, for example, might be meant by a term like androcentric was embarked upon very slowly and dimly, sometimes resentfully, as some savage on the fringe of civilization might awkwardly rediscover the wheel … Nevertheless, the notion of ‘‘solidarity’’ with other women and, indeed, the possibility that female primates generally might confront shared problems was beginning to stir and to raise explicit questions about male–female relations in the animals I studied. (Hrdy 1986/2006, p. 151)

A standpoint theorist would be inclined to point out that there were women studying primatology prior to Hrdy. What made Hrdy different was her exposure to the developing feminist consciousness.

This is only a brief sketch of a rich branch of feminist scholarship and there have been significant developments as these positions (for updated surveys see Intemann, 2010; Grasswick, 2018), but it suffices for an understanding of why this stream of thought leads to focusing on the negative influences of industry involvement in science. To begin with, industry involvement with science almost necessarily infuses commercial values into scientific inquiry. Given a view of objectivity that requires scientific inquiry to be disinterested, industry involvement is inherently a source of bias. Thus, as Intemann and de Melo-Martin (2014) have argued, for philosophers who are coming to these issues afresh, feminist epistemology “seems particularly well situated to provide resources to help address such concerns because this literature has both 1) theorized about how to minimize biases in science, e.g., sexist or androcentric biases, and 2) generated accounts of objectivity that do not require individual scientists to be value-neutral or disinterested” (p. 135).

However, coming to look at industry-funded science with the tools of feminist epistemology almost necessarily results in focusing on the perils of industry-funded science. Though it is oversimplifying to a degree, a dominant form of a research project in feminist epistemology is to begin with a piece of accepted science, to next demonstrate how such research was distorted by the infusion of sexist values, and to finally use this distortion to probe the functioning of science. This pattern is repeated when Longino’s framework is applied to commercial applications. For example, Justin Biddle (2007) takes the Vioxx debacle and argues that it was caused by institutional failures at Merck. Internally, numerous researchers at Merck raised red flags regarding the safety data years prior to its removal, yet Merck publicly maintained that Vioxx was safe. Given Merck’s vast ability to shape the scientific literature, Biddle argues that it is implausible to think that adding a final stage of critical discussion, will render objective knowledge.

In all cases that I am aware of this general pattern is repeated: specifically, the philosopher begins with a case where industry influence is seen to be problematic and then applies feminist theories of objectivity to assess whether they are adequate to account for the epistemic failing. Other examples of this pattern include: manufacturing uncertainty regarding the safety risks of commercial products (Borgerson, 2011); the failure to develop a HPV vaccine that can be successfully used in developing nations (de Melo-Martin and Intemann, 2011), the distortion of the science on the health effects of second-hand smoke (Fernandez Pinto, 2014), distortion of the reliability of anthropogenic climate change (Fernandez Pinto, 2014; Rolin, 2017); a distortion in the agenda of medical research away from illnesses of the poor (Intemann and de Melo-Martin, 2014); the downplaying of the risks that SSRIs induce suicide (Jukola, 2015a); the manipulation of the FDA in the approval of flibanserin for “hypoactive sexual desire disorder” in women (Holman and Geislar, 2018; Bueter and Jukola, 2020).

To be clear, I am neither disputing any of the particular conclusions of the research cited above nor taking issue with the line of research more generally. The point is simply this: logically speaking the framework supplied by feminist epistemology could be used in the analysis of the successful generation of knowledge with industry-academic collaboration, but it never is. A reason for this one-sided focus on the perils of industry funding is that the framework of feminist epistemology is dispositionally critical of existing power structures. This is particularly true of standpoint epistemologies which “require adopting a normative commitment to examining scientific phenomena in ways that challenge, rather than reinforce, systems of oppression” (Intemann and de Melo-Martin, 2014, p. 144). Such a project necessarily focusses on where power corrupts rather than where it refines.

#### The Weaponization of Science

A second intellectual stream by which philosophers of science have come to study the role of industry-funding is by studying what might be called regulatory science. Starting with medical research and food safety in the early 20th century and subsequently with environmental and safety regulations in the mid-twentieth century, national governments attempted to use science to inform public policy and to control and regulate corporate actors.

Though she is less frequently cited amongst philosophers, Kristen Shrader-Frechette is an early and influential example of such a philosopher. Shrader-Frachette’s works include critiques of scientific technology assessment Shrader-Frechette (1980), environmental regulation Shrader-Frechette (1982), risk assessment Shrader-Frechette (1988), and nuclear waste Shrader-Frechette (1993) to name just few representative examples of an voluminous body of work that is primary (and atypically) aimed outside of the philosophical discourse.^5^ More proximally influential has been the work of Naomi Oreskes and Eric Conway’s historical work on how a small group of industry-sponsored scientists were able to derail or delay significant legislation of smoking, second-hand smoke, acid rain, ozone depletion, and global warming. A similar vein of research emerges from the study of the Tobacco Industry and agnotology—the intentional production of ignorance (Proctor 1995; 2008; 2011; Fernandez Pinto 2015; 2017). Finally, medical research and particularly the manipulation of scientific research by the pharmaceutical industry has been a similar entry point for a number of philosophers now working on the topic (Brown, 2002, 2004, 2008; Biddle, 2007; Jukola, 2015a, 2015b, 2017; Holman, 2015, 2019; Holman and Bruner, 2015; Stegenga, 2018).

What is of crucial importance is that what has drawn philosopher’s attention has been only the portion of industry-funded science that interface with regulatory spheres where science is designed to constrain otherwise profitable activity. Indeed, often times profit-seeking entities are asked to conduct scientific studies on their own products. This creates an incentive to satisfy the letter of the law with regards to producing scientific evidence, while conducting studies which produce results which are systematically skewed in favor of their commercial interests. Simultaneously, regulators (and other reformers) are incentivized to revise governmental structure and scientific methodology in ways that prevent or remediate distortions introduced by the regulated entity. Because these pressures remain in place on both parties and because profit incentives and truth frequently diverge, science becomes the battleground for an asymmetric arms race between the two parties (Holman, 2015; Holman and Geislar, 2018).

Yet while the regulatory sphere creates incentives for industry to distort science, there are several other domains where, at least on a prima facie basis, it seems that market incentives and reliable science align. For example, the oil and gas industry has funded a significant amount of scientific research that it uses for exploration. Similarly, the pharmaceutical industry conducts or funds a large amount of R&D work in the process of drug development that is not immediately concerned with regulatory approval or promotion. Yet with a few exceptions (e.g., Adam, 2005), this has garnered almost no attention from philosophers of science. Accordingly, this selective focus on some industry science but not others has left philosophers with a distorted view of the whole.

#### The Epochal Break

A final, though less central vein has brought a third group of philosophers to study the role of industry on science. Though it is less central, it is worth mentioning here because it is a direct response to the very administrators who are the consumers of research policy scholarship. As Willem Halffman and Hans Radder (2015) write in *The Academic Manifesto: From an Occupied University*:

The university has been occupied … [by] a mercenary army of professional administrators, armed with spreadsheets, output indicators, and audit procedures … the scientific publication system is now all but broken: it is caving in under an endless stream of worthless publications, edited papers posing as republications ‘for a different audience’, strategic citations, and opportunistic or commercial journals: an exponentially growing stream of output, hardly ever read. You do not further your career in this publication factory (Halffman and Leydesdorff, 2010; Abma, 2013) by *reading* all these papers, but rather by *writing* as many as possible, or at least by adding your name to them—and finding this absolutely normal (p. 165ff).

The primary concern is that the very nature of the university is changing in a self-reinforcing cycle with the nature of science from a mission of public service to a mission for private enrichment. Such concerns are variously expressed as both concerns for the university itself and for character of science conducted within it (Krimsky, 2004; Carrier, Howard, and Kourany, 2008 [esp. part 3]; Carrier and Nordmann, 2011; Nordmann et al., 2011; Radder, 2010; 2019). On this view the norms of the business world are inherently corrosive to the mission of academic scholarship. Again, it is no surprise that scholars brought to the topic from this approach tend to focus on the drawbacks of industry-funded science generally and industry-academic partnerships in particular.

## Discussion: Synergy or Schism

In the course of this paper I have summarized two very different bodies of literature on the effects of industry-funding on scientific research. I have argued that each of the bodies of research remains partial, and as such has no claim as a definitive assessment of the full scope of the phenomenon. In closing, I wish to sketch out how the two bodies of work might be fruitfully integrated.

First, while it may first appear that the two literatures are bereft of overlap, there are some potential points of contact. The first is the effect of patents on scholarship and the changes brought about to encourage technology transfers from the university to the private sector. The research policy literature find that researchers who are involved in patenting publish both more and a higher quality of research (Agrawal and Henderson 2002; Azoulay et al., 2007; Breschi et al., 2007; Fabrizo and Di Minin, 2008). Moreover, researchers report a perception that patenting increases their professional reputation (Owen-Smith and Powell, 2001; Moutinho et al., 2007; van Rijnsoever et al., 2008). In contrast, philosophers have argued that patenting can produce “thickets” which prohibit the rest of the community from engaging in work in the area (Sterckx 2010; Biddle, 2014). These are not inherently contradictory, it is conceivable, for example, that patents allow a small handful of researchers to corner some area of study allowing them to produce significant research to the detriment of the remainder of the scientific community. Exploring this and contrasting views on legislation like the Bayh-Dole act seems like a particularly fruitful area for further work (representative philosophical work includes Irzık, 2007; Brown, 2008; Biddle, 2011; Sterckx 2011; Biddle, 2015; for representative work from research policy scholars see Thursby and Thursby, 2002; Sampat et al., 2003; Mowery and Sampat, 2004; Powers and McDougall, 2005).

Beyond areas where the literatures could be immediately integrated, each body of scholarship poses a direct challenge to the other. Accordingly, it should be productive for each literature to apply its methods to cases that run against the general dispositions of the discipline. Specifically, with regard to policy research, we might ask how its standard outcome measures function in an area of science that we currently have good reason to think was epistemically defective.

For example, we have in-depth accounts of numerous drug disasters, such as the development and promotion of anti-arrhythmic drugs (Moore, 1995). In broad strokes, we know that a group of researchers collaborating with industry was able to produce research that anti-arrhythmic medications were safe in the face of a group of more senior researchers who raised serious concerns about drug safety (Holman, 2019). This occurred in large part because industry actively sought out scholars whose research methods would best portray their products and funded their research (Holman and Bruner, 2017) while actively blacklisting researchers who raised concerns (Moore, 1995).

Importantly, in this case, not only were the drugs dangerous, researchers were in a position to know they were dangerous and had it not been for the influence of industry, it is very likely that such drugs would never have been used. Though histories of this period provide the broad strokes of how research unfolded, an elaborated account could be paired with how policy research metrics faired. For example, it might be that the epistemically inferior studies were published in lower-tier journals and that the high prevalence of usage was due to promotion that occurred outside of medical journals. Alternatively, it could be that industry funding increased both the quantity of publications as well as the venues that those studies were published. The former scenario would be one in which the metrics used by policy researchers correctly gauge high-quality science, while the latter would show a worrisome detachment of productivity metrics from genuine scientific contributions. If we find case after case where researchers who engage with industry succeed on productivity metrics but produce epistemically defective research, then we begin to undermine the claim that such measures track the production of scientific knowledge.

Similarly, revisiting the different streams that brought industry-funding to the attention of philosophers of science shows ways in which some of their efforts might be profitably diverted. Feminist philosophers of science have focused on where the power of industry has distorted scientific inquiry or undermined its integrity, but where financial interests and truth align shouldn’t we expect science to be advanced? Surely, the discovery and swift production of COVID-19 vaccines provide an example of the tremendous benefit of industry science generally and in some cases the collaboration of industry-university collaborations (e.g., the partnership between Oxford and AstraZeneca).

By the same token philosophers of science could make detailed case studies of industry-funded science that is not specifically targeted towards regulatory ends. As indicated above, regulatory science provides the vast majority of the cases discussed by philosophers of science (Holman and Elliott, 2018), but barely rates a mention in the survey of industry-academic engagement (Perkmann et al., 2021). This strongly suggests that philosophers of science have an overly narrow and unrepresentative sample of the industry-funded science. Moreover, it is likely to be the case that inquiry outside areas of regulatory impact function neither like areas with regulatory impact nor like university-based science. What little work has been done here suggests that science is conducted in ways that are different than university science, but which nevertheless produce genuine scientific advances (Adam et al., 2006).

Finally, for those concerned with the “epochal break” and the changing nature of the university, there would seem to be a new group of scholars receptive to such engagement. Science policy scholars have recently identified “socially engaged” universities as a promising area for future research (Fecher and Friesike, 2014; Grau et al., 2017), but the literatures have evolved in isolation from the discussion in philosophy of science. As elsewhere, science policy scholars have identified areas where philosophers have been overly gloomy. For example, Perkmann et al. (2019) have investigated purportedly successful cases where the ideals and norms of academic research were shielded from the negative effects of industry engagement. These domains seem like a natural place to begin to integrate and address the concerns expressed by those who advocate for public-interest science (Krimsky, 2004; Carrier et al., 2008 [esp. part 3]; Carrier and Nordman, 2011; Nordmann et al., 2011; Radder, 2010; 2019).

In short, both disciplines have blind spots. In my view, part of the explanation for why research policy scholars are entirely unconcerned about the influence of industry on scientific research is that they lack the tools to detect any problems. The philosophy of science literature suggests there are significant problems to detect if a means of measurement could be devised. Yet the general negativity with which philosophers view the influence of industry stands in stark contrast with views expressed by many who are so engaged. It is possible that such researchers are deluded; however, I have also offered a reason why the research agendas that have brought philosophers of science to study these issues predispose them to focus on the perils of industry-funded science. I expect that the same close attention to the promise of industry-funded science could open up a significant amount of rich intellectual ground and go some way to explain why research policy scholars have such an open embrace of industry-academic relations.

## Data Availability

The original contributions presented in the study are included in the article/Supplementary Material, further inquiries can be directed to the corresponding authors.

## Footnotes

1 In this section, I am only referring to philosophers of science. There have been other scholars including medical journal editors (e.g., Angell, 2004), medical researchers (e.g, Glantz et al., 1998), and environmental policy scholars (e.g., Krimsky, 2004), that have also written on industry funding and science and tend to be critical of it. These contributions could be seen as policy research, though it does not fit the narrow definition used in this paper (see note 3). To some extent, the literature within the philosophy of science is integrated into this larger body of work, but policy research is not. Tracing the histories of this diverse array of scholarship is left as a project for another day.

2 In using “distort” rather than a more neutral term such as “shape”, I am intentionally taking over the value-laden tone of this literature.

3 I am here using “policy research” in a narrow sense to refer to the discipline as it generally conceives of itself and particularly as the discipline is captured in the summary papers of Perkmann et al. (2013, 2021). There is a broader sense in which many scholars conduct work that is relevant to science policy, including for example, many of the scholars cited in *The Perils of Industry Funding in Science*. My discussion of policy research in this paper is directed at this narrow sense.

4 With regard to the first two streams. I have separated them out, but philosophers whose primary interest is within one, frequently find themselves writing about the other. Accordingly, they might also be seen as two tributaries of the same stream.

5 It should be noted that a group of practically oriented philosophers grew up around Notre Dame including Don Howard and Janet Kourany. This group collaborated with Martin Carrier at Bielefeld. Within the field, a number of the scholars now working on the topic studied emerged from Notre Dame (Kevin Elliott, Justin Biddle, Manuela Fernandez Pinto, and Chris ChoGlueck) or Bielefeld (Torsten Wilholt).
